# FOXO3a protects glioma cells against temozolomide-induced DNA double strand breaks via promotion of BNIP3-mediated mitophagy

**DOI:** 10.1038/s41401-021-00663-y

**Published:** 2021-04-20

**Authors:** Chuan He, Shan Lu, Xuan-zhong Wang, Chong-cheng Wang, Lei Wang, Shi-peng Liang, Tian-fei Luo, Zhen-chuan Wang, Mei-hua Piao, Guang-fan Chi, Peng-fei Ge

**Affiliations:** 1 Department of Neurosurgery, First Hospital of Jilin University, Changchun, 130021 China; 2 Research Center of Neuroscience, First Hospital of Jilin University, Changchun, 130021 China; 3 Department of Neurology, First Hospital of Jilin University, Changchun, 130021 China; 4 Department of Anesthesiology, First hospital of Jilin University, Changchun, 130021 China; 5 Key Laboratory of Pathobiology, Ministry of Education, Jilin University, Changchun, 130021 China

**Keywords:** glioma, temozolomide, DNA double strand break, oxidative stress, mitochondrial superoxide, BNIP3, FOXO3a, mitophagy, 3MA, bafilomycin A

## Abstract

FOXO3a (forkhead box transcription factor 3a) is involved in regulating multiple biological processes in cancer cells. BNIP3 (Bcl-2/adenovirus E1B 19-kDa-interacting protein 3) is a receptor accounting for priming damaged mitochondria for autophagic removal. In this study we investigated the role of FOXO3a in regulating the sensitivity of glioma cells to temozolomide (TMZ) and its relationship with BNIP3-mediated mitophagy. We showed that TMZ dosage-dependently inhibited the viability of human U87, U251, T98G, LN18 and rat C6 glioma cells with IC_50_ values of 135.75, 128.26, 142.65, 155.73 and 111.60 μM, respectively. In U87 and U251 cells, TMZ (200 μM) induced DNA double strand breaks (DSBs) and nuclear translocation of apoptosis inducing factor (AIF), which was accompanied by BNIP3-mediated mitophagy and FOXO3a accumulation in nucleus. TMZ treatment induced intracellular ROS accumulation in U87 and U251 cells *via* enhancing mitochondrial superoxide, which not only contributed to DNA DSBs and exacerbated mitochondrial dysfunction, but also upregulated FOXO3a expression. Knockdown of FOXO3a aggravated TMZ-induced DNA DSBs and mitochondrial damage, as well as glioma cell death. TMZ treatment not only upregulated BNIP3 and activated autophagy, but also triggered mitophagy by prompting BNIP3 translocation to mitochondria and reinforcing BNIP3 interaction with LC3BII. Inhibition of mitophagy by knocking down BNIP3 with SiRNA or blocking autophagy with 3MA or bafilomycin A1 exacerbated mitochondrial superoxide and intracellular ROS accumulation. Moreover, FOXO3a knockdown inhibited TMZ-induced BNIP3 upregulation and autophagy activation. In addition, we showed that treatment with TMZ (100 mg·kg^−1^·d^−1^, ip) for 12 days in C6 cell xenograft mice markedly inhibited tumor growth accompanied by inducing FOXO3a upregulation, oxidative stress and BNIP3-mediated mitophagy in tumor tissues. These results demonstrate that FOXO3a attenuates temozolomide-induced DNA double strand breaks in human glioma cells via promoting BNIP3-mediated mitophagy.

## Introduction

Glioma is the most common primary malignant brain tumor with a high modality and rate of recurrence [1]. The current standard of care for newly diagnosed glioma includes surgery, radiotherapy and adjuvant chemotherapy with temozolomide (TMZ) [2]. As a widely used oral alkylating agent, TMZ is more effective than any other chemotherapeutic drug in prolonging the median survival of glioma patients [2, 3]. Although multiple factors, such as upregulated expression of MGMT and multidrug resistance proteins, were reported to limit the toxicity of TMZ in glioma cells [3, 4], emerging evidence has revealed that autophagy plays a crucial role in protecting glioma cells against TMZ treatment [5, 6].

Autophagy is an evolutionarily conserved catabolic process that maintains cellular homeostasis by degrading unneeded cellular materials or dysfunctional organelles [7]. Autophagic removal of damaged mitochondria is designated mitophagy, which is morphologically characterized by the formation of mitophagosomes in the cytoplasm [8]. During the course of mitophagy, the mitochondria destined for removal are engulfed into autophagosomes to form mitophagosomes. Then, these mitophagosomes fuse with lysosomes to form mitolysosomes, in which mitochondria are degraded by lysosomal enzymes [8]. Autophagic removal of impaired mitochondria could inhibit intracellular accumulation of reactive oxygen species (ROS) and prevent mitochondrial release of pro-death factors such as AIF and cytochrome *c* [9]. Although autophagy confers protection to glioma cells against TMZ-induced death [5, 6], it remains unclear whether this protection depends on the removal of damaged mitochondria.

Priming damaged mitochondria is a key step leading to mitophagy, although mitochondrial fragmentation following the loss of the mitochondrial membrane potential often precedes mitophagy. In addition to NIX and FUNDC1, BNIP3 (Bcl-2/adenovirus E1B 19-kDa-interacting protein 3) is a receptor that primes damaged mitochondria for autophagic removal [8]. BNIP3-mediated mitophagy is involved in multiple physiological and pathological processes, such as limiting the glycolytic shift in radioresistant cancer, promoting the differentiation of cardiac progenitor cells and rescuing renal cells from ischemic injury [10–12]. Endothelial monocyte-activating polypeptide-II was found to enhance TMZ toxicity in glioma stem cells by inducing BNIP3-mediated mitophagy [13], but it remains elusive whether TMZ alone could trigger mitophagy in glioma cells.

FOXO3a (forkhead box transcription factor 3a) is a member of the FOX family. As a transcription factor, FOXO3a regulates multiple cellular functions, such as cell proliferation, differentiation, invasive migration and cell death [14]. A clinical study showed that FOXO3a expression is upregulated with glioblastoma progression and could be used to predict poor patient prognosis [15]. Experimental data showed that FOXO3a promoted glioma cell resistance to TMZ by causing nuclear accumulation of β-catenin [16]. However, FOXO3a plays dual roles in the regulation of autophagy in cancer cells. FOXO3A not only regulates doxorubicin-induced protective autophagy in hepatocellular carcinoma cells [17] but also contributes to brazilin-induced lethal autophagy in osteosarcoma cells [18]. Notably, it was reported to participate in regulating tunicamycin-induced BNIP3 upregulation in cardiomyocytes [19]. Although these previous studies suggested that autophagy activation and BNIP3 expression are both regulated by FOXO3a, it is necessary to clarify whether FOXO3a contributes to BNIP3-mediated mitophagy. Therefore, in this study, we investigated the role of FOXO3a in TMZ-induced glioma cell death and its relationship with BNIP3-mediated mitophagy.

## Materials and Methods

### Reagents

Temozolomide (TMZ), glutathione (GSH), metalloporphyrin Mn(III) tetrakis(4-benzoic acid)porphyrin (MnTBAP), and anti-LC3B antibodies were purchased from Sigma (St. Louis, MO). TMZ was dissolved in DMSO to a storage concentration of 50 mmol/L. Primary antibodies against the following proteins were purchased from Abcam (Cambridge, MA): FOXO3a, BNIP3, ATG5, p62 (SQSTM1), AIF, TOMM20, COXIV, VDAC1, phospho-H2AX at Ser139, HIF-α and H2A. Anti-β-Actin antibody was purchased from Santa Cruz Biotechnology (Santa Cruz, CA). Other reagents were purchased from Sigma (St. Louis, MO).

### Glioma cell lines and culture

Human U87, U251, T98G, and LN18 glioma cells and rat C6 glioma cells were all obtained from the Shanghai Institute of Cell Biology, Chinese Academy of Sciences (Shanghai, China). They were cultured in DMEM supplemented with 10% fetal bovine serum, 2 mmol/L glutamine, penicillin (100 U/mL) and streptomycin (100 μg/mL) and maintained at 37 °C and 5% CO_2_ in a humidified environment. Cells in the exponential growth phase were used in the experiments.

### Cellular viability and cell death assays

Cellular viability was assessed using an MTT assay and was expressed as a percentage of the absorbance value at 562 nm of the control cells. Cell death was assessed by using a lactate dehydrogenase cytotoxicity assay kit according to the manufacturer’s instructions (Beyotime Biotech, Nanjing, China), and the absorbance value of each sample was read at 490 nm. The cell death percentage was calculated by using the following formula: cell death percentage (%) = (*A*sample − *A*control)/(*A*max − *A*control) × 100, where *A*sample is the sample absorbance value; *A*control is the absorbance value of the control group; and max is the absorbance value of the positive group.

### Mitochondrial membrane potential assays

The mitochondrial membrane potential was assayed by using JC-1 staining (Beyotime Biotech, Nanjing, China) according to the manufacturer’s instructions. The collected cells were analyzed by flow cytometry (FACScan, Becton Dickinson, San Jose, CA).

### Measurement of intracellular ROS, mitochondrial superoxide and H_2_O_2_

Intracellular ROS was evaluated by using DCFH-DA (Beyotime Biotech, Nanjing, China) according to the manufacturer’s instructions. The green fluorescence intensity was measured at an excitation wavelength of 485 nm and an emission wavelength of 528 nm using a fluorescence spectrometer (HTS 7000, Perkin Elmer, Boston, MA). The ROS levels were expressed as arbitrary units/mg protein and then as a percentage of the control. Mitochondrial superoxide was assayed by using MitoSOX red according to the manufacturer’s instructions (Invitrogen, Eugene, OR). The red fluorescence intensity was measured at an excitation wavelength of 530 nm and an emission wavelength at 590 nm and was expressed as a ratio to the fluorescence in control cells. Cells were seeded on 6-well plates, stained with DCFH-DA or MitoSox red as described above and then observed under a fluorescence microscope (Olympus IX71, Tokyo, Japan).

H_2_O_2_ levels were analyzed with an H_2_O_2_ assay kit (Beyotime Biotech, Nanjing, China) according to the manufacturer’s protocol. Briefly, 10 mg of glioma tissues was added to lysis buffer, homogenized, and centrifuged to obtain the supernatant. Then, 50 μL of supernatant and 100 μL of test solution were added to a tube at room temperature for 30 min and measured with a microplate reader at a wavelength of 560 nm. Absorbance values were calibrated to a standard concentration curve to calculate the concentration of H_2_O_2_. Finally, the results were expressed as a percentage of the concentration relative to that in the control cells.

### Transfection of small interfering RNA (SiRNA)

The cells were seeded onto a culture dish. Transfection of siRNA was performed by using Lipofectamine 2000 (Invitrogen, Eugene, OR) according to the manufacturer’s instructions. siRNAs targeting BNIP3 (5′- GAUUACUUCUGAGCUUGCATT-3′), FOXO3a (5′-CGUGAUGCUUCGCAAUGAUTT-3′), and ATG5 (5′-GACGUUGGUAACUGACAAATT-3′) as well as scrambled siRNA (5′-UUCUCCGAACGUGUCACGUTT-3′) were purchased from GenePharma Company (Suzhou, China). After transfection of siRNA overnight, the cells were incubated with TMZ for the indicated times for subsequent experiments.

### StubRFP-SensGFP-LC3 assay and immunocytochemical staining

The StubRFP-SensGFP-LC3 assay was performed according to the manufacturer’s instructions (GeneChem, Shanghai, China). Briefly, U87 cells (5 × 10^4^ cells/mL) seeded in a culture dish were incubated with StubRFP-SensGFP-LC3B lentivirus (MOI = 5 × 10^6^ TU/mL). The medium was changed after 12 h of incubation. After infection for 72 h, the cells were treated with TMZ at the indicated dosage for the indicated time and then treated with MitoTracker deep red (Invitrogen company, Eugene, OR). Finally, the cells were observed under a confocal laser scanning microscope (Olympus FV3000, Tokyo, Japan).

Then, U87 cells seeded in a culture dish were incubated in the presence or absence of TMZ at the indicated dosage for the indicated times before they were treated with 100 nmol/L MitoTracker red (Invitrogen Company, Eugene, OR) for 30 min at 37 °C before fixation in ethanol. After the nonspecific antibody binding sites were blocked, the cells were incubated with anti-BNIP3 (1:100) followed by Alexa Fluor 488-conjugated goat anti-rabbit IgG (1:200) for 1 h. Finally, the cells were visualized under a laser scanning confocal microscope (Olympus FV3000, Tokyo, Japan).

### Transmission electron microscopy

After the cells were harvested with 0.25% trypsin and then washed with PBS, they were collected by centrifugation for 10 min at 2000 revolutions per minute. Then, the cells were fixed in ice-cold 2.5% glutaraldehyde in PBS (pH 7.3), rinsed with PBS, postfixed in 1% osmium tetroxide with 0.1% potassium ferricyanide, dehydrated through a graded series of ethanol (30%–90%) and embedded in Epon resin (Energy Beam Sciences, Agawam, MA, USA). Semithin (300 nm) sections were obtained using a Reichart Ultracut ultramicrotome, stained with 0.5% toluidine blue and examined under a light microscope. Ultrathin sections (65 nm) were stained with 1% uranyl acetate and 0.1% lead citrate and examined under a JEM2000EX transmission electron microscope (JEOL, Pleasanton, CA, USA).

### Rat C6 tumor xenograft in mice

Athymic BALB/c nude mice (age 4 weeks, weight 20–22 g, Beijing Vital River Laboratory Animal Technology Company, China) were housed in a specific pathogen-free environment under a 12-h light/12-h dark cycle, provided free access to food and water, and acclimatized to their surroundings for three days. The mice were cared for in accordance with the guidelines for experimental animals of Jilin University, and the study was approved by the Ethics Committee of the First Hospital of Jilin University (Changchun, China). A total of 1 × 10^7^ C6 cells in the exponential growth phase in 100 μL of PBS were subcutaneously injected into the right flank of each mouse. Therapeutic experiments were started when the tumor reached approximately 150 mm^3^ (~7 days). The mice were allocated to receive daily intraperitoneal injections of vehicle (*n* = 10/group) or the same volume of 100 mg/kg body weight TMZ for 12 days (*n* = 10/group). The tumor size was measured using a slide caliper, and the tumor volume was calculated using the formula 0.5 × A × B^2^, in which A is the tumor length and B is the tumor width. The day after the last treatment, the mice were euthanized by cervical dislocation. After being excised and weighed, the tumors were immediately frozen in liquid nitrogen for Western blotting analysis.

### Gel electrophoresis and Western blotting

The glioma cells collected by centrifugation and frozen xenografted glioma tissues were homogenized with a glass Pyrex microhomogenizer (20 strokes) in ice-cold lysis buffer (Beyotime Biotech, Nanjing, China). Homogenates were centrifuged at 800 × *g* for 10 min at 4 °C to obtain supernatant 1 and pellet 1. Supernatant 1 was then centrifuged at 10,000 × *g* for 10 min at 4 °C to obtain supernatant 2 and pellet 2. Pellet 1 was the nuclear fraction, pellet 2 was the mitochondrial fraction, and supernatant 2 was the cytoplasmic fraction. The protein content was determined using a Bio-Rad protein assay kit. After the proteins were subjected to SDS electrophoresis and transferred to PVDF membranes, the membranes were blocked with 3% BSA in TBS for 30 min at room temperature and then incubated overnight at 4 °C with primary antibodies. Next, the membranes were incubated with horseradish peroxidase-conjugated secondary antibody and washed, and the immunoreactive proteins were visualized on a chemiluminescence developer (ChemiScope 5300, Clinx Science Instrument Company, Shanghai).

### Coimmunoprecipitation

U87 and U251 glioma cells were collected by centrifugation following harvesting with a scraper, and the removed tumors were homogenized as described above and then centrifuged at 15,000 × *g* for 15 min at 4 °C to obtain the supernatant. The protein content was determined using a Bio-Rad protein assay kit, and the protein concentrations were normalized. Protein samples (400 μg) were precleared with isotype IgG control antibody (Abcam) and Protein A/G agarose (Millipore). Protein A/G agarose (40 μL) incubated with 10 μL primary antibody in 50 μL lysis buffer overnight at 4 °C was added to the protein samples and then incubated overnight at 4 °C. The mixture was precipitated by high-speed centrifugation at 12,000 r/min for 10 s at 4 °C. To eliminate nonspecific binding, the agarose was washed three times with lysis buffer. Agarose-bound immunocomplexes were then released by denaturing solution in loading buffer before Western blot analysis was performed.

### Statistical analysis

All data represent at least 4 independent experiments and are expressed as the means ± SDs. Statistical comparisons were made using one-way ANOVA. *P* values less than 0.05 were considered to represent statistical significance.

## Results

### TMZ-induced DNA DSBs and mitochondrial damage in glioma cells

**Fig. 1:**
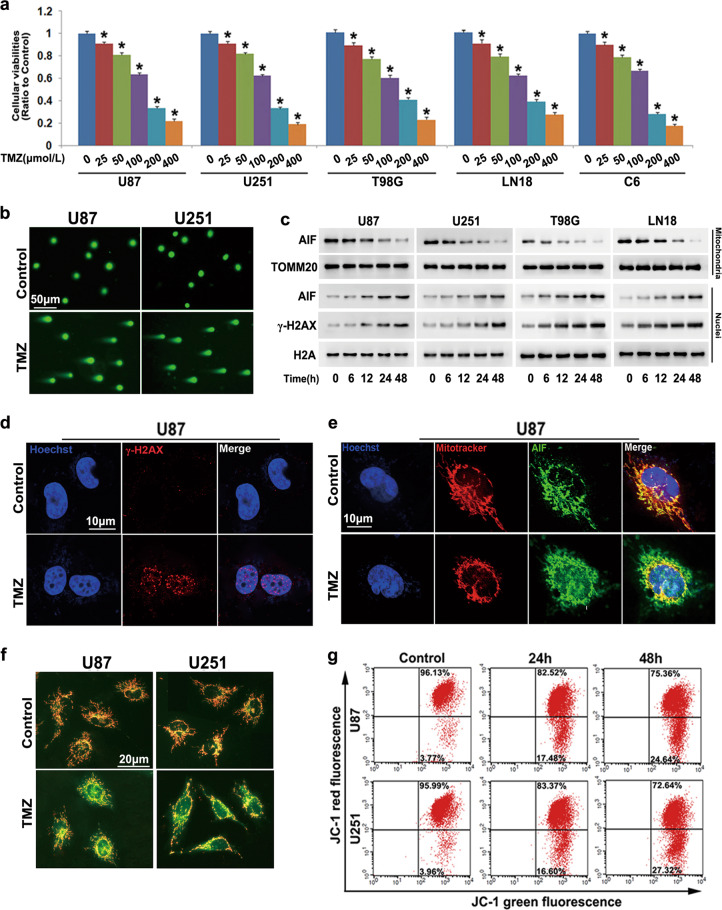
TMZ-induced DNA DSBs and mitochondrial damage in glioma cells. **a** The MTT assay showed that TMZ inhibited the viability of U87, U251, T98G, LN18 and C6 glioma cells in a dose-dependent manner after incubation for 72 h. **b** Neutral comet assay showed that U87 and U251 cells exhibited longer comet tails after treatment with TMZ for 48 h. **c** Western blotting revealed that TMZ treatment induced a reduction in mitochondrial AIF levels but improved nuclear AIF and γ-H2AX levels in a time-dependent manner. **d** Representative images of confocal microscopy combined with immunochemical staining showed that many γ-H2AX foci formed in the nuclei of the U87 cells treated with TMZ for 48 h. **e** Representative images of confocal microscopy combined with immunochemical staining indicated that TMZ-induced AIF accumulation in nuclei after incubation for 48 h. **f** Representative images of JC-1-stained cells under a fluorescence microscope suggested that the red fluorescence exhibited by JC-1 was obviously decreased in TMZ-treated U87 and U251 cells compared with respective control cells. **g** Flow cytometry analysis combined with JC-1 staining showed that TMZ-induced time-dependent depletion of the mitochondrial membrane potential. *P < 0.01 versus control group. The values are expressed as the means ± SEMs (*n* = 5 per group)

To investigate the toxic effect of TMZ on glioma cells, human U87, U251, T98G and LN18 glioma cells and rat C6 glioma cells were treated with TMZ at the indicated concentrations for 72 h, and then cellular viabilities were determined by the MTT assay. We found that TMZ significantly inhibited the viability of glioma cells at a concentration of 25 μmol/L, and this inhibition became more apparent when the TMZ dose was increased to 50, 100, 200 and 400 μmol/L (Fig. 1a). This suggested that TMZ inhibited glioma cell viability in a dose-dependent manner. We thus calculated the IC_50_ values of TMZ at 72 h, and they were 135.75 μmol/L for U87 cells, 128.26 μmol/L for U251 cells, 142.65 μmol/L for T98G cells, 155.73 μmol/L for LN18 cells, and 111.60 μmol/L for C6 cells. Therefore, TMZ at 100 and 200 μmol/L was used in subsequent experiments.

Given that TMZ is an alkylating agent that targets DNA, we performed a neutral comet assay that is specifically used to detect DNA double-strand breaks (DSBs). As shown in Fig. 1b, cells treated with 200 μmol/L TMZ had longer comet tails at 48 h, which was not observed in the control cells. Then, we used Western blotting to analyze TMZ-induced changes in the levels of γ-H2AX (phospho-H2AX at Ser139), which is generated at the sites of DNA DSBs and is thus regarded as a molecular marker of DNA DSBs [20]. γ-H2AX expression was significantly upregulated by TMZ in a time-dependent manner (Fig. 1c). Confocal microscopy combined with immunochemical staining revealed that many foci of γ-H2AX were induced by TMZ and formed at 48 h in the nuclei of U87 cells (Fig. 1d). These results indicated that TMZ-induced DNA DSBs in glioma cells.

Because γ-H2AX could serve as a platform to recruit apoptosis inducing factor (AIF), which acts as a nuclease to cause chromatinolysis when translocated from mitochondria to nuclei, we isolated mitochondrial and nuclear fractions and examined AIF distribution. As the Western blotting results showed, mitochondrial AIF levels were decreased in response to 200 μmol/L TMZ in a time-dependent manner, but nuclear AIF levels increased correspondingly at each indicated time (Fig. 1c). Moreover, confocal microscopy combined with immunochemical staining also confirmed that TMZ treatment promoted AIF accumulation in the nuclei of U87 cells at 48 h (Fig. 1e). These results indicated that TMZ-induced AIF translocation from mitochondria to nuclei in glioma cells.

Considering that AIF release from mitochondria is controlled by the mitochondrial membrane potential [21], we tested whether TMZ-induced depletion of the mitochondrial membrane potential by conducting JC-1 staining. As a fluorescence probe, JC-1 emits red fluorescence when bound as aggregates in healthy mitochondria but emits green fluorescence as a monomer upon release from damaged mitochondria [22]. As revealed by fluorescence microscopy, the red fluorescence exhibited by JC-1 was brighter than the green fluorescence in control U87 and U251 cells but decreased drastically after cells were treated with 200 μmol/L TMZ for 48 h (Fig. 1f). Flow cytometry analysis combined with JC-1 staining showed that the mitochondrial membrane potential was decreased by TMZ at 24 h and further decreased at 48 h (Fig. 1g). These results indicated that TMZ treatment resulted in mitochondrial damage.

Therefore, these results suggested that TMZ treatment not only resulted in DNA damage but also led to mitochondrial dysfunction.

### Damaged mitochondria aggravated TMZ-induced DNA DSBs via increased ROS production

**Fig. 2:**
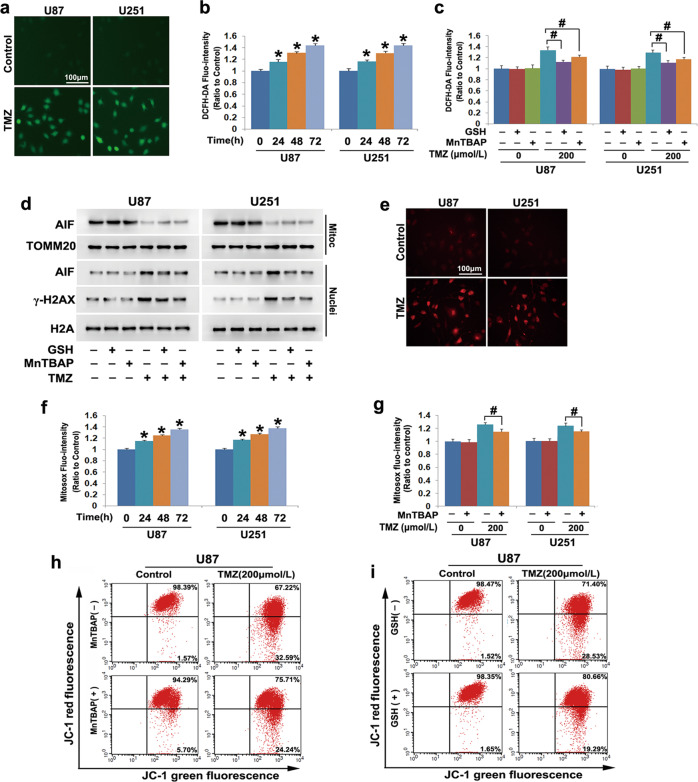
Damaged mitochondria aggravated TMZ-induced DNA DSBs via increases in ROS. **a** Representative images under a fluorescence microscope revealed that the green fluorescence exhibited by DCFH-DA was much brighter in TMZ-treated cells than in control cells. **b** Statistical analysis showed that the fluorescence intensity increased in a time-dependent manner upon TMZ treatment. **c** TMZ-induced increases in the green fluorescence exhibited by DCFH-DA were obviously abrogated in the presence of GSH or MnTBAP. **d** Western blotting showed that the TMZ-induced reduction in AIF in the mitochondrial fractions and increases of AIF and γ-H2AX in the nuclear fractions were all markedly inhibited by MnTBAP or GSH. **e** Representative images acquired by fluorescence microscopy showed that the red fluorescence exhibited by MitoSOX red was much brighter in TMZ-treated cells than in control cells. **f** Statistical analysis showed that the red fluorescence was increased by TMZ in a time-dependent manner. **g** Pretreatment with MnTBAP significantly alleviated the TMZ-induced increases in mitochondrial superoxide levels. **h** Flow cytometry analysis combined with JC-1 staining showed that TMZ-induced depletion of the mitochondrial membrane potential was blocked by MnTBAP. **i** Flow cytometry analysis combined with JC-1 staining showed that pretreatment with GSH prevented TMZ-induced mitochondrial depolarization. *P < 0.01 versus the control group; ^#^*P* < 0.01 between the indicated two groups. The values are expressed as the means ± SEMs (*n* = 5 per group)

Because DNA DSBs could be induced under conditions of oxidative stress [20], we examined the role of ROS in TMZ-induced DNA DSBs by using the fluorescent ROS probe DCFH-DA. As the fluorescence microscopy images show, the green fluorescence was much brighter in the cells treated with 200 μmol/L TMZ for 48 h than in control cells (Fig. 2a). Statistical analysis also indicated that the fluorescence intensity was significantly increased by TMZ after 24 h of treatment and further increased after 48 h and 72 h of treatment (Fig. 2b). These results indicated that TMZ-induced time-dependent accumulation of intracellular ROS in glioma cells. In contrast, pretreatment with the antioxidant GSH at 10 mmol/L for 1 h not only significantly suppressed TMZ-induced ROS (Fig. 2c) but also obviously inhibited γ-H2AX formation (Fig. 2d and Supplementary Fig. 1a). Moreover, flow cytometry combined with Annexin V and PI double staining revealed that TMZ-induced glioma cell death was clearly inhibited by GSH (Supplementary Fig. 1b). These results indicated that TMZ-induced ROS-dependent DNA DSBs.

To clarify the source of TMZ-induced ROS, we examined mitochondrial superoxide levels by using the fluorescence probe MitoSOX red. Fluorescence microscopy showed that the cells treated with 200 μmol/L TMZ exhibited stronger red fluorescence at 48 h than did control cells (Fig. 2e). This was also supported by statistical data showing that the fluorescence intensity was obviously increased after TMZ treatment for 24 h and further enhanced when the treatment time was extended to 48 h and 72 h (Fig. 2f). In contrast, pretreatment with the mitochondrial superoxide inhibitor MnTBAP at 40 μmol/L for 1 h not only significantly inhibited the TMZ-induced increases in mitochondrial superoxide levels (Fig. 2g) but also prevented the accumulation of intracellular ROS (Fig. 2c, d). Correspondingly, TMZ-induced γ-H2AX formation was also prevented by MnTBAP treatment (Fig. 2d and Supplementary Fig. 1a). Flow cytometry of Annexin V and PI double staining showed that TMZ-induced glioma cell death was markedly inhibited in the presence of MnTBAP (Supplementary Fig. 1b). These results indicated that mitochondrial damage contributed to TMZ-induced DNA DSBs via increased intracellular ROS levels.

Notably, TMZ-induced mitochondrial depolarization and nuclear translocation of AIF were obviously alleviated in the presence of MnTBAP (Fig. 2h, d, and Supplementary Fig. 1c, d). These results indicated that TMZ-induced mitochondrial damage and the nuclear translocation of AIF by increasing mitochondrial superoxide levels. Furthermore, pretreatment with GSH obviously inhibited TMZ-induced mitochondrial depolarization and the nuclear translocation of AIF (Fig. 2i, d, and Supplementary Fig. 1c, d). These results indicated that ROS originating from TMZ-damaged mitochondria could reversibly exacerbate mitochondrial dysfunction or impair other healthy mitochondria. Therefore, mitochondrial damage plays a crucial role in the regulation of TMZ-induced glioma cell death.

### Autophagy inhibited TMZ-induced oxidative stress

**Fig. 3:**
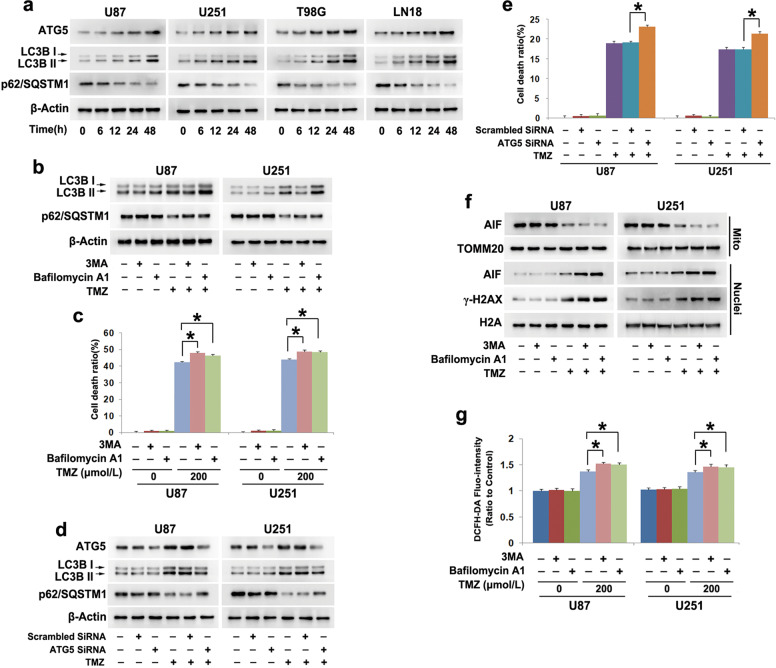
Autophagy inhibited TMZ-induced oxidative stress in glioma cells. **a** Western blotting showed that TMZ-triggered time-dependent increases in ATG5 and LC3BII expression but reductions in the autophagy substrate p62 (SQSTM1). **b** Western blotting revealed that pretreatment with 3MA obviously inhibited the TMZ-induced increase in LC3BII levels and reduction in p62 (SQSTM1) levels. Bafilomycin A1 reversed the TMZ-induced reduction in p62 (SQSTM1) but reinforced the upregulation of LC3BII expression. **c** The LDH release assay showed that TMZ-induced glioma cell death was exacerbated in the presence of 3MA or bafilomycin A1. **d** Western blotting revealed that knockdown of ATG5 with siRNA reversed TMZ-induced upregulation of LC3BII expression and reduction in p62 (SQSTM1) expression. **e** The LDH release assay revealed that TMZ-induced glioma cell death was exacerbated when ATG5 was knocked down with siRNA. **f** Western blotting analysis demonstrated that TMZ-induced γH2AX formation and AIF nuclear translocation were inhibited by 3MA or bafilomycin A1. **g** Statistical analysis of the fluorescence exhibited by DCFH-DA demonstrated that pretreatment with 3MA or bafilomycin A1 obviously reinforced TMZ-induced increases in ROS. *P < 0.01 between indicated two groups. The values are expressed as the means ± SEMs (*n* = 5 per group)

Although autophagy has been reported to protect glioma cells against TMZ-induced death [5, 6], its underlying mechanism still needs to be elucidated. Thus, we examined TMZ-induced changes in autophagy-related marker proteins by using Western blotting. It was found that 200 μmol/L TMZ-induced time-dependent upregulation of ATG5 and LC3BII expression and increases in the LC3BII and LC3BI ratio (Fig. 3a and Supplementary Fig. 2a, b) as well as a corresponding reduction in the levels of the autophagy substrate p62 (SQSTM1) at each indicated time (Fig. 3a and Supplementary Fig. 2c).

To address the role of autophagy in TMZ-induced glioma cell death, U87 and U251 cells were treated for 1 h with the autophagy initiation inhibitor 3MA at 5 mmol/L or the autophagy flux inhibitor bafilomycin A1 at 1.5 μmol/L before they were incubated with TMZ at 200 μmol/L for 48 h. Western blotting showed that the TMZ-induced increases in both LC3BII protein levels and the ratio between LC3BII and LC3BI and the decrease in p62 (SQSTM1) levels were all partially alleviated by 3MA (Fig. 3b and Supplementary Fig. 2d, e). Although bafilomycin A1 reversed the TMZ-induced reduction in p62 (SQSTM1), it reinforced the upregulation of LC3BII protein levels and the ratio between LC3BII and LC3BI (Fig. 3b and Supplementary Fig. 2d, e). LDH release assays confirmed that glioma cell death caused by 200 μmol/L TMZ was exacerbated by 3MA or bafilomycin A1 (Fig. 3c). Moreover, flow cytometry of Annexin V and PI double staining showed that TMZ-induced glioma cell death was obviously enhanced by 3MA or bafilomycin A1 (Supplementary Fig. 2f). Thus, these results indicated that autophagy prevented TMZ-induced glioma cell death. Consistently, we found that knockdown of ATG5 with siRNA not only prevented TMZ-induced upregulation of both LC3BII protein levels and the ratio between LC3BII and LC3BI but also reversed the reduction in p62 (SQSTM1) levels (Fig. 3d and Supplementary Fig. 2g–i). Moreover, ATF5 knockdown exacerbated glioma cell death (Fig. 3e). This further verified that autophagy conferred protection to glioma cells against TMZ-induced death.

Then, Western blotting showed that TMZ-induced AIF nuclear translocation and γ-H2AX formation were both reinforced when the cells were pretreated with 3MA and bafilomycin A1 (Fig. 3f and Supplementary Fig. 2j–l). Since ROS contribute to TMZ-induced mitochondrial damage and DNA DSBs, we examined TMZ-induced ROS levels when autophagy was inhibited. The TMZ-mediated improvement in intracellular ROS was obviously strengthened by 3MA or bafilomycin A1 (Fig. 3g). These results indicated that autophagy inhibited TMZ-induced mitochondrial damage and DNA DSBs via mitigation of intracellular ROS.

### TMZ-activated mitophagy in glioma cells

**Fig. 4:**
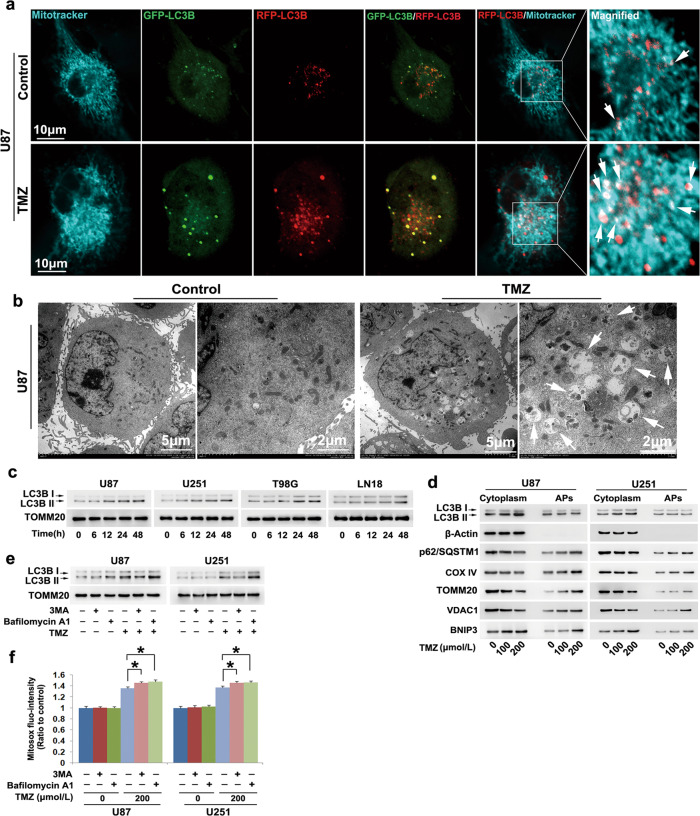
TMZ activated mitophagy in glioma cells. **a** Representative confocal microscopy images of U87 cells transinfected with stubRFP-sensGFP-LC3B lentiviruses showed that mitochondria presented tubular structures and mutual connections, but they became fragmented in the cells treated with 200 μmol/L TMZ for 48 h. Moreover, many more green puncta and red puncta formed in the cytoplasm of TMZ -treated cells than in control cells. The green puncta colocalized with parts of the red puncta to exhibit a yellow color, indicating that autophagosomes and autolysosomes were both induced by TMZ. Moreover, parts of the red puncta colocalized with fragmented mitochondria (arrowheads), suggesting that TMZ-induced autophagosomes to envelop damaged mitochondria. **b** Transmission electronic microscopy revealed many vacuoles containing mitochondria-like structures (arrowheads) in TMZ-treated cells but not in control cells. **c** Western blotting showed that TMZ improved LC3BII levels in mitochondrial fractions in a time-dependent manner. **d** Western blotting analysis of autophagosomes (APs) pulled down with LC3B antibody showed that there was no significant difference in the level of LC3BII immunoprecipitated with LC3B antibody between control cells and TMZ-treated cells. The levels of autophagy substrate p62 (SQSTM1) and the mitochondrial marker proteins COXIV, TOMM20 and VDAC1 were all obviously increased by TMZ in the APs pulled down by LC3B antibody, although their levels were decreased in the cytoplasmic fractions. **e** Western blotting showed that TMZ-induced upregulation of LC3BII in the mitochondrial fraction was inhibited by 3MA but reinforced by bafilomycin A1. **f** Statistical analysis of the fluorescence exhibited by MitoSOX red showed that the TMZ-induced improvement in mitochondrial superoxide levels was enhanced in the presence of 3MA or bafilomycin A1. *P < 0.01 between the indicated two groups. The values are expressed as the means ± SEMs (*n* = 5 per group)

To address why autophagy could inhibit TMZ-induced oxidative stress, U87 cells transinfected with stubRFP-sensGFP-LC3B lentiviruses were used to examine whether TMZ-induced mitophagy, which could inhibit intracellular ROS by clearing damaged mitochondria [9]. As revealed by confocal microscopy combined with MitoTracker deep red staining, mitochondria in control cells presented tubular structures and were mutually connected. However, they became disordered and even fragmented into spherical forms when cells were incubated with 200 μmol/L TMZ for 48 h (Fig. 4a). This indicated that TMZ triggered mitochondrial fragmentation. It was also found that many green puncta and red puncta were induced by TMZ to form in the cytoplasm, and the green puncta colocalized with parts of the red puncta to exhibit yellow color. Because green fluorescence is prone to quenching in an acidic environment, the red puncta reflect autolysosomes, and the yellow puncta reflect autophagosomes. This indicated that TMZ activated autophagy flux. Moreover, parts of the red puncta also colocalized with fragmented mitochondria (arrowheads), indicating that the damaged mitochondria were engulfed in autophagosomes. Consistently, transmission electronic microscopy showed that many vacuoles containing mitochondria-like structures (arrowheads) could be found in the cytoplasm of TMZ-treated cells but not of control cells (Fig. 4b). Moreover, Western blotting revealed that LC3BII and the ratio between LC3BII and LC3BI were both significantly increased in the mitochondrial fractions by TMZ in a time-dependent manner (Fig. 4c and Supplementary Fig. 3a). These results indicated that TMZ induced the formation of mitophagosomes or mitolysosomes in glioma cells in a time-dependent manner.

To further verify that mitochondria were engulfed in autophagosomes, we used an LC3B antibody to isolate autophagosomes and examined the expression of mitochondrial marker proteins by Western blotting. No significant difference could be found in the protein level of LC3BII in the autophagosomes isolated with LC3B antibody between control cells and TMZ-treated cells, although LC3BII expression and the ratio between LC3BII and LC3BI were both obviously increased by TMZ in the cytoplasmic fractions (Fig. 4d and Supplementary Fig. 3b). Then, we found that autophagy substrate p62 (SQSTM1) and mitochondrial marker proteins COXIV, TOMM20 and VDAC1 were all obviously enriched in autophagosomes (Fig. 4d and Supplementary Fig. 3d), although their levels were decreased in the cytoplasmic fractions upon TMZ treatment (Fig. 4d and Supplementary Fig. 3d). Moreover, this improvement became more apparent when the TMZ dose was increased from 100 μmol/L to 200 μmol/L. Therefore, these results indirectly suggested that TMZ triggered the formation of mitophagosomes in glioma cells. To test whether TMZ-induced activation of mitophagy, we examined the mitochondrial level of LC3BII in the presence of 3MA or bafilomycin A1 and found that TMZ-induced increases in LC3BII levels and the ratio between LC3BII and LC3BI in mitochondrial fractions were both inhibited by 3MA but reinforced by bafilomycin A1 (Fig. 4e, and Supplementary Fig. 3e). Moreover, TMZ-induced mitochondrial superoxide production was also reinforced in the presence of 3MA or bafilomycin A1 (Fig. 4f). This suggested that TMZ induced mitophagy activation, which inhibits oxidative stress by degrading mitochondria with higher levels of superoxide.

### BNIP3 promoted TMZ-induced mitophagy by priming mitochondria

**Fig. 5:**
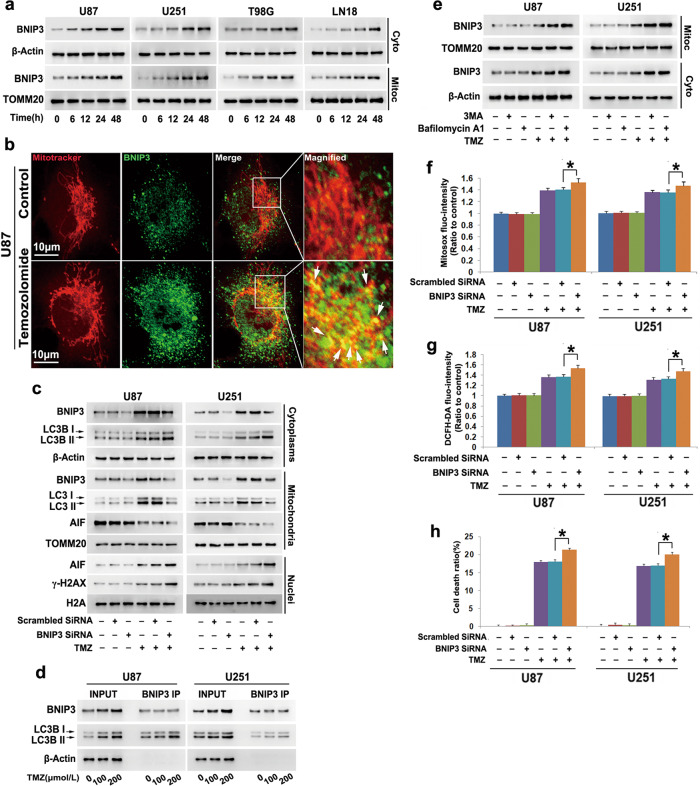
BNIP3 promoted TMZ-induced mitophagy by priming mitochondria. **a** Western blotting showed that TMZ-induced BNIP3 upregulation in both the cytoplasmic and mitochondrial fractions in a time-dependent manner. **b** Representative images acquired by confocal microscopy combined with immunocytochemistry revealed that BNIP3 (green) expression was not only upregulated by TMZ but also obviously induced to colocalize with mitochondria (red) compared with that in control cells. **c** Western blotting revealed that knockdown of BNIP3 with siRNA reinforced TMZ-induced upregulation of LC3BII in the cytoplasmic fraction but inhibited TMZ-triggered improvement of LC3BII in the mitochondrial fraction. Concomitantly, TMZ-induced γH2AX formation and nuclear translocation of AIF were both enhanced. **d** Immunoprecipitation using a BNIP3 antibody indicated that LC3BII coimmunoprecipitated with BNIP3, which became apparent when the TMZ dose was increased from 100 μmol/L to 200 μmol/L. **e** Western blotting showed that TMZ-induced BNIP3 upregulation in the cytoplasmic and mitochondrial fractions was enhanced in the presence of 3MA or bafilomycin A1. **f** Statistical analysis of the fluorescence exhibited by MitoSOX red demonstrated that BNIP3 knockdown enhanced the TMZ-triggered increases in mitochondrial superoxide. **g** Statistical analysis of DCFH-DA fluorescence proved that the TMZ-induced improvement in ROS was strengthened when BNIP3 was knocked down with siRNA. **h** LDH release assays showed that knockdown of BNIP3 exacerbated TMZ-induced glioma cell death. *P < 0.01 between indicated two groups. The values are expressed as the mean ± SEM (*n* = 5 per group)

Considering that BNIP3 could prime damaged mitochondria for autophagic removal [8], we used Western blotting to investigate TMZ-induced changes in BNIP3 expression. Compared with control cells, cells treated with 200 μmol/L TMZ showed time-dependent increases in BNIP3 in the mitochondrial fraction and cytoplasmic fraction (Fig. 5a). Confocal microscopy combined with MitoTracker red staining revealed that BNIP3 (green) was obviously colocalized with mitochondria (red) after 48 h of incubation with TMZ (Fig. 5b). These results indicated that TMZ not only upregulated the expression of BNIP3 but also promoted BNIP3 translocation to mitochondria.

To clarify the role of BNIP3 in TMZ-induced mitophagy, siRNA was introduced to knock down BNIP3. When compared with the cells transfected with scrambled siRNA, cells transfected with BNIP3-specific siRNA showed obvious inhibition of TMZ-induced BNIP3 upregulation in the cytoplasmic and mitochondrial fractions (Fig. 5c). Then, we found that knockdown of BNIP3 inhibited the TMZ-induced improvement in LC3BII levels in the mitochondrial fractions but further improved the LC3BII level in the cytoplasmic fraction (Fig. 5c). This indicated that BNIP3 might recruit autophagosomes to mitochondria. Thus, immunoprecipitation was used to test whether TMZ could enhance the interaction between BNIP3 and autophagosomes. Although there was no significant difference in the level of BNIP3 immunoprecipitated by its antibody between TMZ-treated cells and control cells, the amount of LC3BII coimmunoprecipitated with BNIP3 increased significantly with an increasing TMZ dose (Fig. 5d). Notably, BNIP3 expression was also increased in autophagosomes isolated by LC3B antibody from TMZ-treated cells (Fig. 4d and Supplementary Fig. 3c). These results indicated that the interaction between BNIP3 and autophagosomes was enhanced by TMZ and that BNIP3 primed mitochondria to be engulfed by autophagosomes. Moreover, blocking autophagy with 3MA or bafilomycin A1 obviously reinforced TMZ-induced BNIP3 increases in both the mitochondrial and cytoplasmic fractions (Fig. 5e). These results indicated that BNIP3 could be degraded with mitochondria via the autophagy pathway.

Then, we tested whether knockdown of BNIP3 with siRNA could produce the same effects as autophagy blockade. BNIP3 knockdown significantly aggravated the TMZ-induced improvement in mitochondrial superoxide and intracellular ROS levels (Fig. 5f, g). Correspondingly, TMZ-induced glioma cell death, nuclear translocation of AIF and upregulation of γ-H2AX were all enhanced when BNIP3 was knocked down (Fig. 5h, c). These findings were consistent with the results when autophagy was inhibited with 3MA or bafilomycin A1 and further indicated that BNIP3-mediated mitophagy prevented TMZ-induced glioma cell death.

### FOXO3a contributed to TMZ-induced mitophagy

**Fig. 6:**
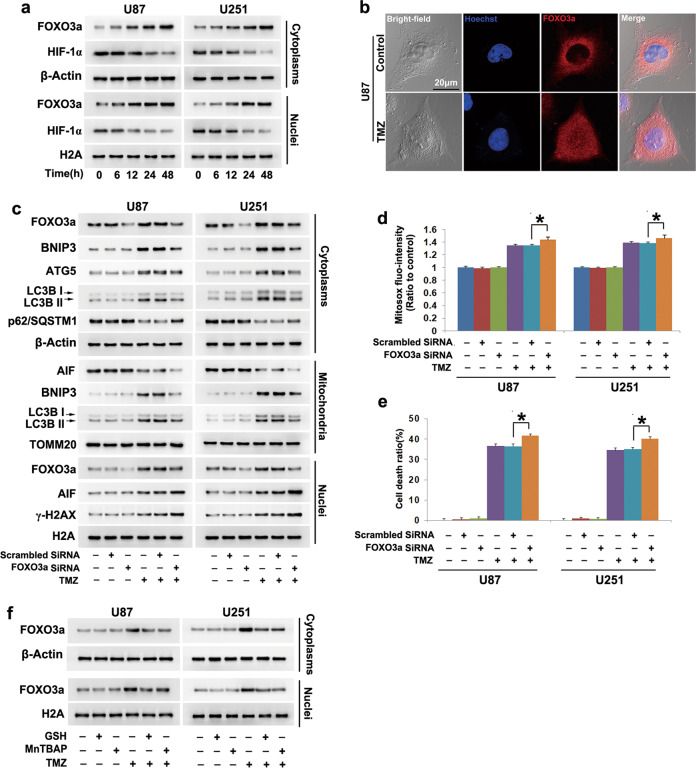
FOXO3a contributed to TMZ-induced BNIP3-mediated mitophagy. **a** Western blotting showed that TMZ-induced time-dependent upregulation of FOXO3a expression and downregulation of HIF-α expression in the cytoplasmic and nuclear fractions. **b** Representative images acquired by confocal microscopy showed that TMZ upregulated the expression of FOXO3a in the nucleus. **c** Knockdown of FOXO3a with siRNA not only inhibited TMZ-induced upregulation of BNIP3 and LC3BII expression in both the cytoplasmic and mitochondrial fractions but also alleviated TMZ-triggered upregulation of ATG5 expression and downregulation of p62 (SQSTM1) expression. Moreover, FOXO3a knockdown enhanced TMZ-induced γ-H2AX formation and nuclear translocation of AIF. **d** Statistical analysis of the fluorescence exhibited by MitoSOX red demonstrated that FOXO3a knockdown enhanced the TMZ-triggered increases in mitochondrial superoxide levels. **e** The LDH release assay showed that knockdown of FOXO3a exacerbated TMZ-induced glioma cell death. **f** Western blotting showed that TMZ-induced upregulation of FOXO3a expression in the cytoplasmic and nuclear fractions was inhibited in the presence of GSH or MnTBAP. *P < 0.01 between indicated two groups. The values are expressed as the means ± SEMs (*n* = 5 per group)

To address why TMZ could trigger mitophagy, Western blotting was used to examine TMZ-induced changes in the transcription factors FOXO3a and HIF-1α, both of which were found to be upstream regulators of mitophagy [23, 24]. Compared with control cells, TMZ-treated U87 and U251 cells exhibited upregulated FOXO3a expression but downregulated HIF-1α expression in a time-dependent manner in (Fig. 6a). Moreover, confocal microscopy demonstrated that TMZ treatment resulted in increased FOXO3a levels in nuclei (Fig. 6b). Notably, we found that FOXO3a expression was markedly upregulated in U87TR cells that were resistant to 200 μmol/L TMZ compared with parental U87 cells (Supplementary Fig. 2m). To clarify the role of FOXO3a in TMZ-induced changes in BNIP3 and autophagy-related marker proteins, we introduced siRNA to knock down BNIP3. As Western blotting showed, knockdown of FOXO3a with siRNA not only obviously prevented TMZ-induced overexpression of BNIP3 but also reversed TMZ-induced increases in ATG5 and LC3II levels and reductions in p62 levels (SQSTM1). Correspondingly, the improvements in BNIP3 and LC3II expression in the mitochondrial fractions were also inhibited (Fig. 6c). Therefore, these results indicated that FOXO3a contributed to TMZ-induced mitophagy. In contrast, TMZ-triggered γ-H2AX formation, nuclear translocation of AIF, and accumulation of mitochondrial superoxide were all enhanced when FOXO3a was knocked down with siRNA (Fig. 6c, d). Consistently, FOXO3a knockdown obviously exacerbated TMZ-induced glioma cell death (Fig. 6e). These results indicated that FOXO3a contributed to TMZ-induced BNIP3-mediated mitophagy.

Given that FOXO3a could be upregulated under oxidative stress [25], we investigated whether oxidative stress contributed to TMZ-induced upregulation of FOXO3a levels in nuclei. When compared with the cells treated with TMZ alone, cells pretreated with GSH or MnTBAP showed obvious inhibition of FOXO3a upregulation (Fig. 6f). These data indicated that oxidative stress promoted TMZ-induced upregulation and nuclear localization of FOXO3a.

### TMZ-induced mitophagy, FOXO3a upregulation and oxidative stress in vivo

**Fig. 7:**
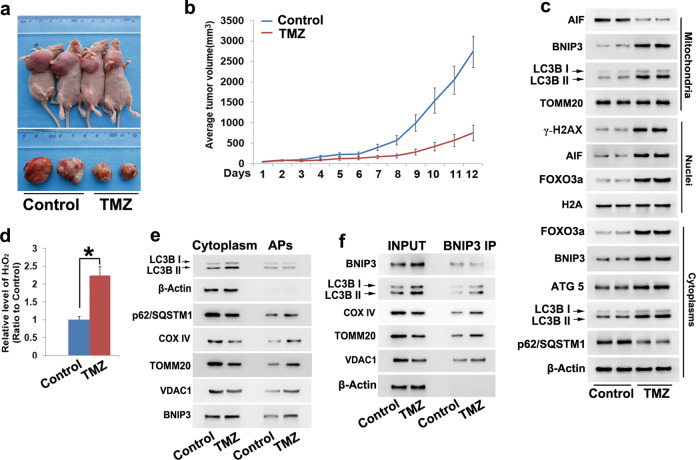
TMZ-induced FOXO3a upregulation and BNIP3-mediated mitophagy in vivo. **a** Representative images of nude mice with xenografted gliomas showed that tumor growth was significantly inhibited when the mice were treated with TMZ at a dose of 100 mg/kg for 12 consecutive days. **b** Statistical analysis of the tumor volumes confirmed that TMZ inhibited tumor growth in vivo. **c** Western blotting showed that TMZ-induced upregulation of FOXO3a and ATG5 expression, increases in BNIP3 and LC3BII levels in the cytoplasmic and mitochondrial fractions, nuclear translocation of AIF and γ-H2AX formation in vivo. **d** Hydrogen peroxide assay showed that TMZ induced the accumulation of hydrogen peroxide in vivo. **e** Western blotting analysis of the autophagosomes (APs) pulled down with LC3B antibody showed that there was no significant difference in LC3BII expression between the control group and TMZ-treated group. However, autophagy substrate p62 (SQSTM1) and the mitochondrial marker proteins COX IV, TOMM20 and VDAC1 all showed obvious increases in expression upon TMZ treatment in the APs isolated with LC3B antibody, although their levels were decreased in the cytoplasmic fractions. **f** Immunoprecipitation with an antibody against BNIP3 revealed that the autophagosome marker LC3BII and mitochondria marker proteins COX IV, TOMM20 and VDAC all obviously coimmunoprecipitated with BNIP3 in the TMZ-treated group. *P < 0.01 between indicated two groups. The values are expressed as the mean ± SEM (*n* = 5 per group)

To examine TMZ toxicity on glioma cells in vivo, C6 cells were xenografted into the right flank of nude mice. Treatment with TMZ at a dose of 100 mg/kg for 12 consecutive days obviously inhibited tumor growth (Fig. 7a), which was also confirmed by statistical analysis of the tumor volumes (Fig. 7b). Western blotting revealed that TMZ treatment not only obviously increased the protein levels of AIF and γ-H2AX in the nuclear fractions but also significantly decreased mitochondrial levels of AIF (Fig. 7c). Additionally, FOXO3a and H_2_O_2_ levels were obviously increased in the TMZ-treated group compared with the control group (Fig. 7c, d). These results indicated that TMZ inhibited the growth of glioma and induced AIF nuclear translocation, γ-H2AX formation, FOXO3a upregulation and oxidative stress in vivo.

Then, we examined whether TMZ-induced mitophagy in glioma cells in vivo. As revealed by Western blotting, the autophagy marker proteins ATG5 and LC3II were both improved, but autophagy substrate p62 (SQSTM1) was decreased in the TMZ-treated group compared with the control group (Fig. 7c). This indicated that TMZ induced autophagy activation in vivo. Then, we used an LC3B antibody to isolate autophagosomes and examined mitochondrial marker protein localization by Western blotting. When compared with the control group, the TMZ-treated group showed decreases in autophagy substrate p62 (SQSTM1) and mitochondrial marker proteins COXIV, TOMM20 and VDAC1 in the cytoplasmic fraction but obviously increases in the autophagosomes isolated by LC3B antibody (Fig. 7e). Thus, these results indicated that TMZ-triggered mitophagy in glioma cells in vivo.

Furthermore, TMZ not only upregulated BNIP3 expression in cytoplasmic fractions but also improved BNIP3 and LC3BII levels in mitochondrial fractions (Fig. 7c). Immunoprecipitation of BNIP3 with its antibody revealed that the autophagosome marker protein LC3BII and the mitochondrial marker proteins COX IV, TOMM20 and VDAC1 all significantly coimmunoprecipitated with BNIP3 in TMZ-treated group (Fig. 7f). Consistently, BNIP3 was also markedly improved in autophagosomes isolated with LC3B antibody (Fig. 7e). These results indicated that mitochondria were primed by BNIP3 and then engulfed by autophagosomes in TMZ-treated gliomas. Thus, TMZ induced BNIP3-mediated mitophagy in vivo.

## Discussion

**Fig. 8:**
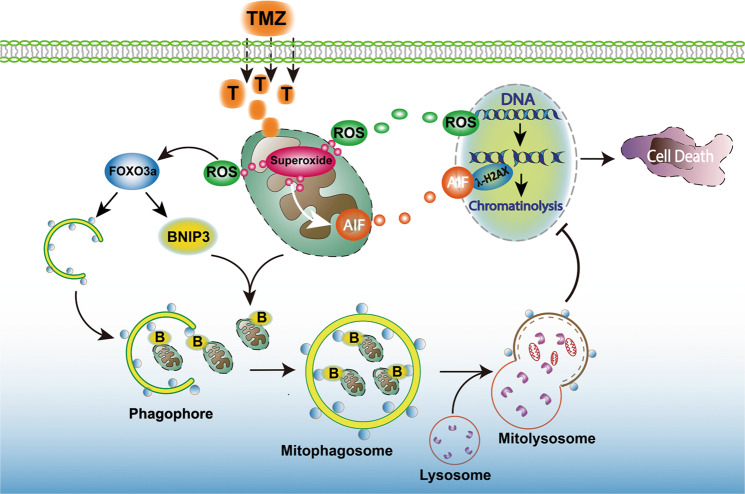
Schematic diagram of the role of FOXO3a in TMZ-induced BNIP3-mediated mitophagy. TMZ treatment induced the excessive generation of mitochondrial superoxide. The overproduction of mitochondrial superoxide not only resulted in mitochondrial depolarization and AIF translocation from mitochondria into nuclei but also led to intracellular accumulation of ROS and ROS-dependent DNA DSBs. Within nuclei, AIF could be recruited to γH2AX that are generated when DNA DSBs occur and could act as a nuclease to degrade DNA (chromatinolysis). Thus, mitochondrial superoxide contributes to TMZ-triggered glioma cell death. On the other hand, the expression of the transcription factor FOXO3a was upregulated by ROS and promoted the expression of BNIP3 and ATG5. BNIP3 was distributed to damaged mitochondria, and ATG5 initiated the formation of phagophores. Then, the mitochondria primed by BNIP3 were engulfed by phagophores to form mitophagosomes, and mitophagosomes fused with lysosomes to form mitolysosomes. Within mitolysosomes, mitochondria are eventually degraded by enzymes released from the lysosomes. Thus, autophagic removal of mitochondria with increased superoxide levels inhibited TMZ-induced glioma cell death via suppression of mitochondria-related oxidative stress. Taken together, these data show that FOXO3a protects glioma cells against temozolomide-induced DNA double-strand breaks via promotion of BNIP3-mediated mitophagy

In summary, we found in this study that TMZ inhibited glioma cell growth and induced DNA DSBs and nuclear translocation of AIF in vitro and in vivo, which was accompanied by BNIP3-mediated mitophagy and FOXO3a upregulation. In vitro studies revealed that TMZ increased ROS by increasing the levels of mitochondrial superoxides, which not only contributed to DNA DSBs and exacerbated mitochondrial dysfunction but also upregulated FOXO3a expression. Knockdown of FOXO3a with siRNA aggravates TMZ-induced DNA DSBs and mitochondrial damage and increases glioma cell death. Mechanistically, TMZ not only activated autophagy and upregulated BNIP3 but also triggered mitophagy by promoting BNIP3 translocation to mitochondria and reinforcing the interaction of BNIP3 with LC3II. Inhibition of mitophagy by knocking down BNIP3 with siRNA or pharmacologically blocking autophagy increased the levels of mitochondrial superoxides and intracellular ROS. Moreover, FOXO3a knockdown obviously inhibited TMZ-induced BNIP3 upregulation and autophagy activation. Therefore, FOXO3a protects glioma cells against TMZ-induced DNA DSBs via promotion of BNIP3-mediated mitophagy (Fig. 8).

Mitochondrial damage has been reported to be involved in multiple types of programmed cell death, such as apoptosis, necroptosis and parthanatos [22, 26, 27]. Damaged mitochondria not only release pro-death factors such as cytochrome *c* and AIF but also increase intracellular ROS levels [28]. It was reported that mitochondrial depolarization and DNA DSBs were both induced when glioma cells were exposed to hydrogen peroxide, a member of the ROS family that can freely penetrate cells [21]. Thus, ROS originating from damaged mitochondria not only impair nuclear DNA but also exacerbate mitochondrial damage. As an alkylating agent targeting DNA, TMZ could also directly impair mitochondria. It was reported that the mitochondria isolated from removed human gliomas swelled and depolarized when exposed to TMZ [29]. Further study revealed that mitochondrial DNA was vulnerable to injury by alkylating agents due to a lack of protective histone proteins [30]. Moreover, TMZ was also reported to inhibit the transcription of the mitochondrial gene ND1 encoding NADH dehydrogenase subunit 1 in complex I and cytochrome B (complex III) [31]. This might explain why TMZ could impair mitochondria: complex I and complex III are primary components of the mitochondrial respiratory chain. In this study, we found that TMZ-induced time-dependent increases in mitochondrial superoxide levels. In contrast, mitigation of mitochondrial superoxide with MnTBAP markedly inhibited TMZ-induced increases in intracellular ROS levels. Suppression of intracellular ROS with GSH not only prevented TMZ-induced γ-H2AX formation but also inhibited mitochondrial depolarization and nuclear translocation of AIF. Therefore, ROS originating from damaged mitochondria contribute to TMZ-induced DNA DSBs and impair other healthy mitochondria or exacerbate mitochondrial damage. Thus, mitochondrial damage also plays a crucial role in TMZ-induced DNA damage and glioma cell death.

Mitophagy is a type of selective autophagy responsible for clearing damaged mitochondria. Thus, its occurrence is dependent on the activation of autophagic flux. Consistent with previous reports [5, 6], we found in this study that TMZ-activated autophagic flux because the TMZ-mediated reduction in the autophagy substrate p62 (SQSTM1) levels was obviously reversed in the presence of 3MA or bafilomycin A1. Moreover, mitophagy is in coordination with mitochondrial fission regulated by Drp1, through which damaged mitochondria can be separated from the mitochondrial network and then engulfed by autophagosomes to form mitophagosomes [32]. Thus, the typical morphological feature of mitophagy is the formation of mitophagosomes in the cytoplasm, which makes it different from other subtypes of autophagy, such as pexophagy, lipophagy and ERphagy [33]. In this study, transmission electronic microscopy revealed that mitochondria-like structures were contained within TMZ-induced vesicles in the cytoplasm. Moreover, confocal microscopy images of U87 cells infected with stubRFP-sensGFP-LC3B lentiviruses combined with MitoTracker deep red staining showed that TMZ-induced autophagosomes colocalized with fragmented mitochondria. These results were consistent with the morphological features of mitophagy [7]. Furthermore, Western blotting revealed that the expression of COXIV, TOMM20 and VDAC1, which are mitochondrial marker proteins, was improved in autophagosomes isolated from TMZ-treated cells by using an LC3B antibody. Thus, TMZ triggered the formation of mitophagosomes in glioma cells. Although mitophagy contributes to glioma cell death induced by compound AT 101 via excessive removal of mitochondria [34], accumulating evidence demonstrates that mitophagy protects cancer cells against chemotherapy. It was reported that mitophagy conferred protection to hepatocellular carcinoma cells against cisplatin-induced apoptosis [35]. In contrast, inhibition of mitophagy sensitized non-small-cell lung cancer A549 cells to the glycolytic inhibitor 3-bromopyruvate [36]. In this study, we found that inhibition of mitophagy by blocking TMZ-activated autophagy with 3MA or bafilomycin A1 increased the mitochondrial accumulation of superoxide and nuclear translocation of AIF, as well as DNA DSBs and glioma cell death. Therefore, TMZ-induced protective mitophagy in glioma cells.

In addition to FUNDC1, which mediates oxidative stress-induced mitophagy in laryngeal cancer cells [37], BNIP3 is also involved in the regulation of mitophagy in cancer cells. It was reported that BNIP3 contributed to ceramide-triggered lethal mitophagy in glioma cells [38]. In contrast, BNIP3-mediated mitophagy was also shown to protect neuroblastoma SH-SY5Y cells against TNF-α-induced death [39]. Thus, these studies showed that BNIP3 could regulate both lethal and protective mitophagy. Previous reports have stated that BNIP3 contributes to mitophagy by activating autophagy and priming mitochondria or the autophagic pathway. On the one hand, it could activate autophagy by binding to Rheb to inhibit mTOR phosphorylation or binding to Bcl-2 to liberate Beclin-1 [8]. On the other hand, it primes impaired mitochondria to be engulfed by autophagosomes, which depends on the insertion of its C-terminal transmembrane domain into the outer membrane of damaged mitochondria and its LC3-interacting regions (LIRs) interacting with LC3B-II [8]. In this study, we found that knockdown of BNIP3 with siRNA abrogated TMZ-induced accumulation of LC3BII on mitochondria but improved LC3BII levels in the cytoplasm. Blocking autophagy reinforced TMZ-induced BNIP3 upregulation in mitochondria. Thus, BNIP3 did not contribute to TMZ-induced autophagy activation but rather recruited autophagosomes to mitochondria. Moreover, BNIP3 knockdown reinforced TMZ-induced increases in mitochondrial superoxide levels and the nuclear translocation of AIF, as well as enhanced intracellular ROS levels and glioma cell death, all of which were consistent with the findings reported when autophagy was blocked. Therefore, BNIP3 contributes to priming dysfunctional mitochondria for autophagic removal in TMZ-induced protective mitophagy.

As a key regulator of cell survival, FOXO3a is involved in the regulation of multiple cellular functions [14] but was also reported to modulate autophagy activation and BNIP3 expression. It contributed to not only doxorubicin-induced protective autophagy in hepatocellular carcinoma cells [17] but also BNIP3 upregulation triggered by tunicamycin in cardiomyocytes [19]. Consistent with this, we found that TMZ-triggered time-dependent upregulation and nuclear translocation of FOXO3a expression. Knockdown of FOXO3a with siRNA markedly prevented TMZ-induced BNIP3 upregulation and autophagy activation and exacerbated glioma cell death. Therefore, FOXO3a contributed to TMZ-induced protective mitophagy mediated by BNIP3. The nuclear localization of FOXO3a is regulated by its phosphorylation status. FOXO3a is phosphorylated by ERK/MAPK at Ser425 and is then retained in the cytoplasm before it is degraded via the ubiquitin-proteasome pathway [40], but phosphorylation of FOXO3a by p38/MAPK at Ser7 improves its nuclear translocation and transcriptional activity [41]. It was reported that TMZ treatment inhibited ERK/MAPK but activated p38/MAPK in glioma cells [42, 43]. Moreover, activated p38/MAPK decreased glioma cell sensitivity to TMZ treatment [43]. Although we did not investigate whether TMZ-induced FOXO3a phosphorylation at Ser7 in this study, we found that the increased nuclear translocation of FOXO3a was obviously inhibited when mitochondrial superoxide was neutralized with MnTBAP or when intracellular ROS were suppressed with GSH. Considering that p38/MAPK activation could occur under oxidative stress [44], we think that ROS promote TMZ-induced upregulation and nuclear translocation of FOXO3a via activation of p38.

In conclusion, we demonstrate in this study that FOXO3a protects glioma cells against TMZ treatment by activating BNIP3-mediated mitophagy, which inhibits the nuclear translocation of AIF and DNA DSBs via inhibition of oxidative stress. Thus, inhibition of FOXO3a is a potential strategy to improve glioma cell sensitivity to TMZ treatment.

## Supplementary information

Supplementary Figure 1

Supplementary Figure 2

Supplementary Figure 3
