# Supplementary figures and images for: FOXO3a protects glioma cells against temozolomide-induced DNA double strand breaks via promotion of BNIP3-mediated mitophagy
Source: Acta Pharmacol Sin. 2021 Apr 20;42(8):1324–37. doi: 10.1038/s41401-021-00663-y (PMC8285492; doi:10.1038/s41401-021-00663-y)

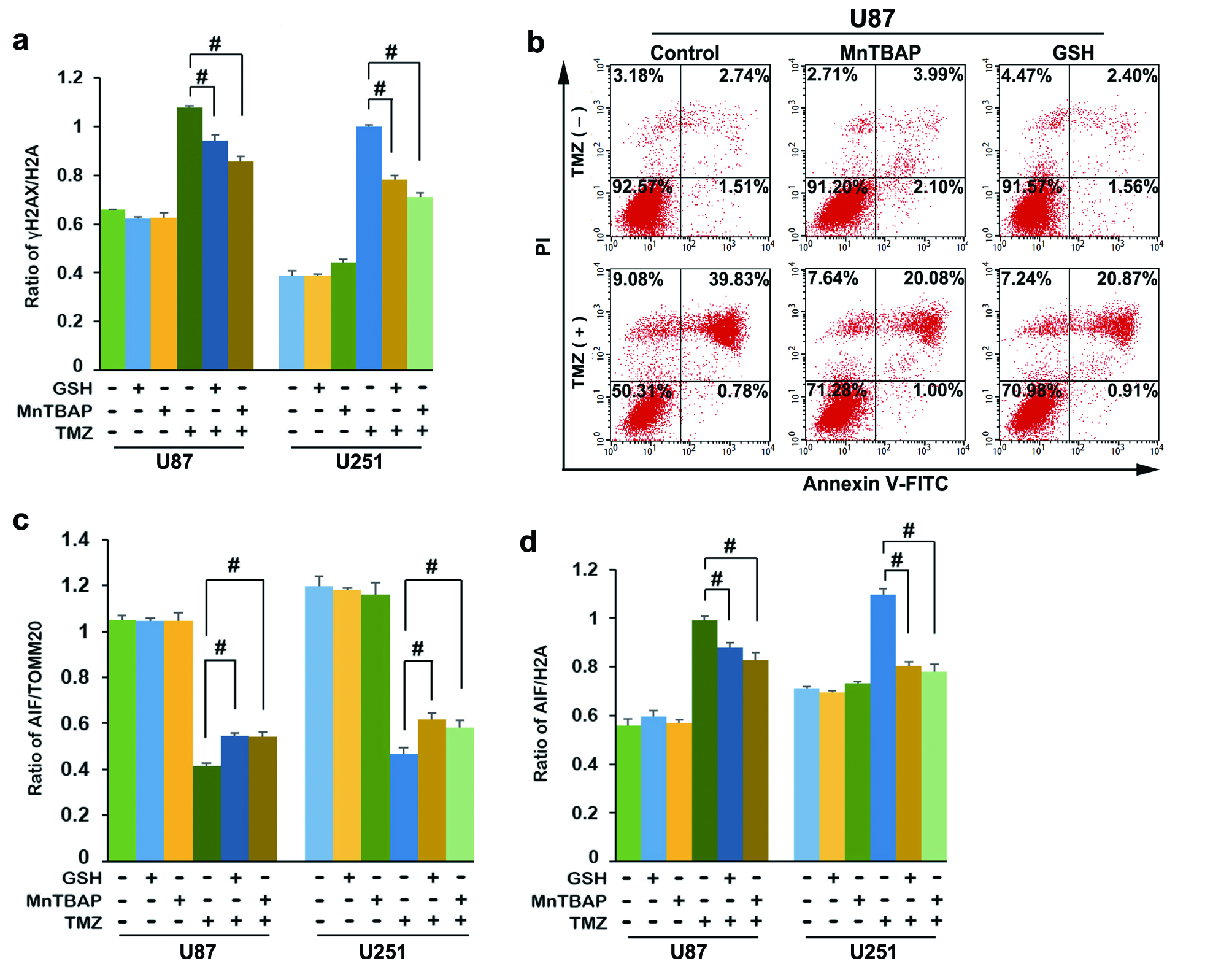

Supplement: Supplementary file 1 — Supplementary Figure 1 [file 41401_2021_663_MOESM1_ESM.tif]

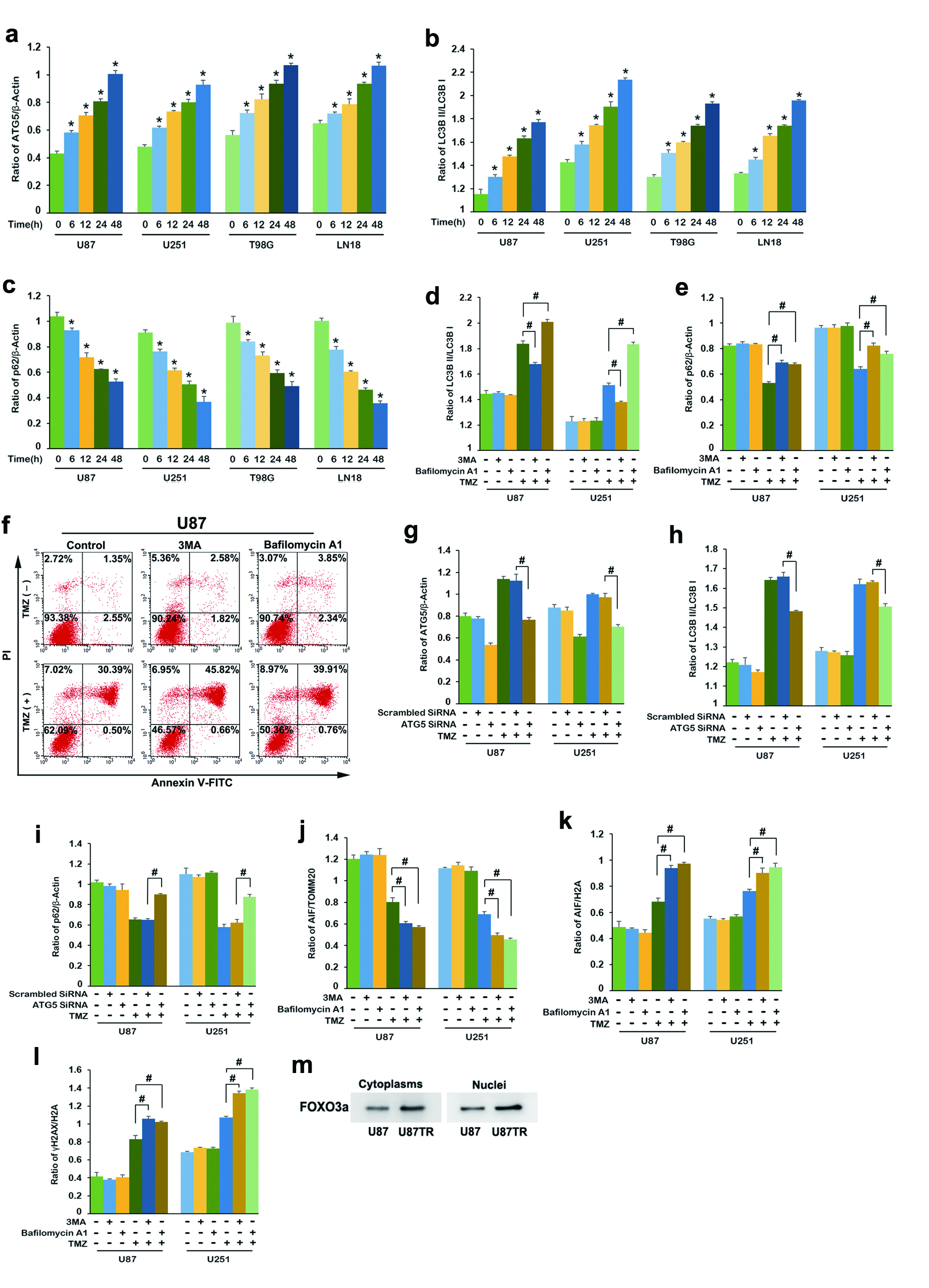

Supplement: Supplementary file 2 — Supplementary Figure 2 [file 41401_2021_663_MOESM2_ESM.tif]

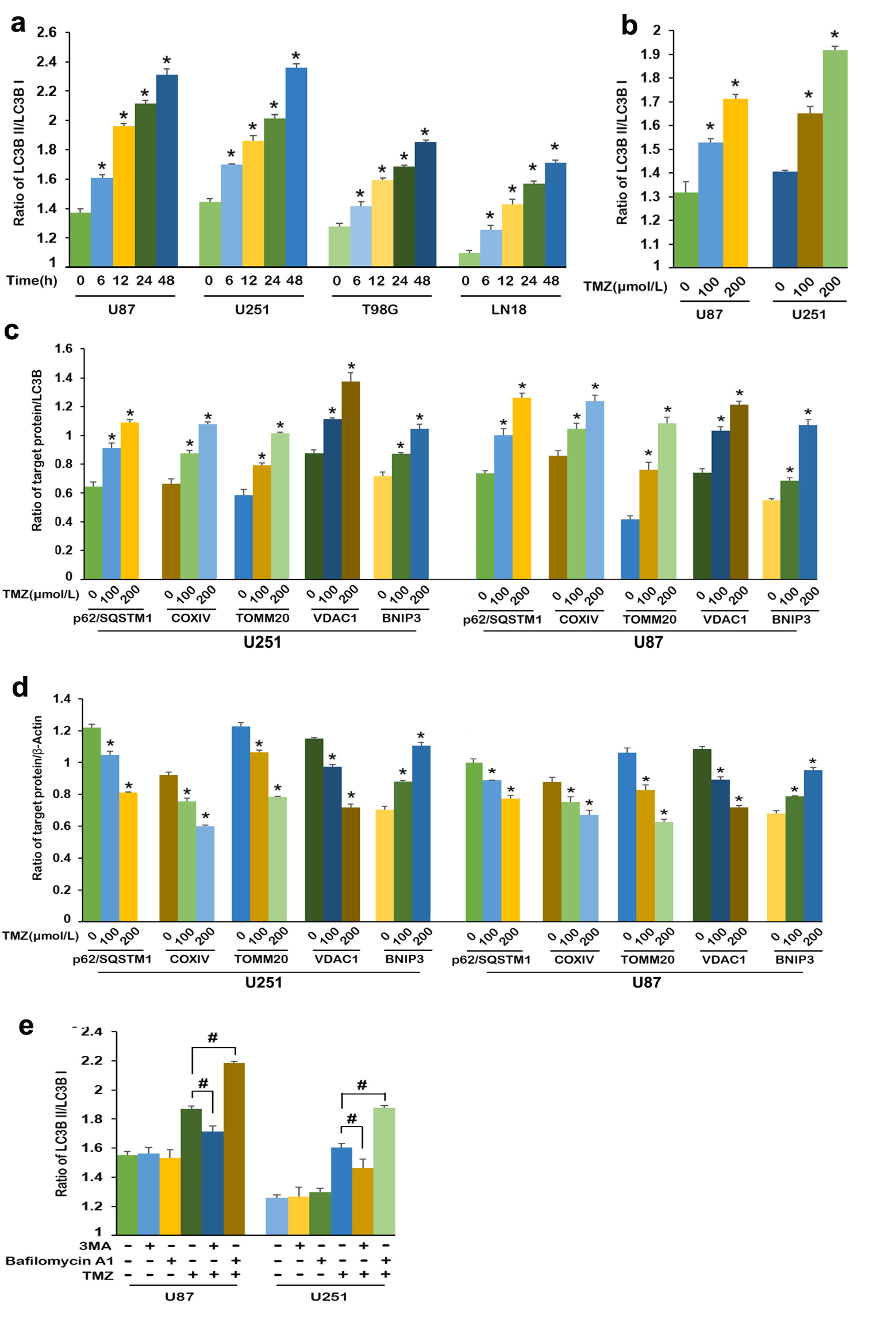

Supplement: Supplementary file 3 — Supplementary Figure 3 [file 41401_2021_663_MOESM3_ESM.tif]
